# Virus-specific memory CD4 T cells sustain long-term numerical and functional impairment following whole-body irradiation

**DOI:** 10.1093/jimmun/vkag244

**Published:** 2026-09-10

**Authors:** Shravan Kumar Kannan, Mohammad Heidarian, Elizabeth A Escue, John T Harty, Vladimir P Badovinac

**Affiliations:** Interdisciplinary Graduate Program in Immunology, Carver College of Medicine, University of Iowa, Iowa City, IA, United States; Department of Pathology, Carver College of Medicine, University of Iowa, Iowa City, IA, United States; Holden Comprehensive Cancer Center, Carver College of Medicine, University of Iowa, Iowa City, IA, United States; Department of Pathology, Carver College of Medicine, University of Iowa, Iowa City, IA, United States; Holden Comprehensive Cancer Center, Carver College of Medicine, University of Iowa, Iowa City, IA, United States; Experimental Pathology Graduate Program, Carver College of Medicine, University of Iowa, Iowa City, IA, United States; Department of Pathology, Carver College of Medicine, University of Iowa, Iowa City, IA, United States; Holden Comprehensive Cancer Center, Carver College of Medicine, University of Iowa, Iowa City, IA, United States; Experimental Pathology Graduate Program, Carver College of Medicine, University of Iowa, Iowa City, IA, United States; Interdisciplinary Graduate Program in Immunology, Carver College of Medicine, University of Iowa, Iowa City, IA, United States; Department of Pathology, Carver College of Medicine, University of Iowa, Iowa City, IA, United States; Experimental Pathology Graduate Program, Carver College of Medicine, University of Iowa, Iowa City, IA, United States; Interdisciplinary Graduate Program in Immunology, Carver College of Medicine, University of Iowa, Iowa City, IA, United States; Department of Pathology, Carver College of Medicine, University of Iowa, Iowa City, IA, United States; Holden Comprehensive Cancer Center, Carver College of Medicine, University of Iowa, Iowa City, IA, United States; Experimental Pathology Graduate Program, Carver College of Medicine, University of Iowa, Iowa City, IA, United States

**Keywords:** intracellular infections, memory CD4 T cells, radiation exposure, T cell function

## Abstract

The increasing demand for nuclear power and radiotherapy has expanded potential avenues for human exposure to whole-body irradiation (WBI), underscoring the need to understand its impact on immune cells. While prior studies have explored radiation-induced disruptions in immune homeostasis, the direct effect of WBI on pre-existing memory CD4 T cells remains elusive. Using T cell receptor transgenic CD4 T cell chimeric mice infected with lymphocytic choriomeningitis virus–Armstrong, we demonstrate that a sublethal WBI dose (5 Gy) causes a significant and persistent reduction in the numbers of memory CD4 T cells for at least 30 d post-WBI. At 8 d post-WBI, memory CD4 T cells exhibited an increased frequency of Ly6C^+^ T helper 1 cells and a decreased frequency of PSGL1^+^Ly6C^−^ central memory cells in the spleen compared with mock-irradiated (0 Gy) control mice. These subset imbalances were no longer detectable by 30 d post-WBI. However, surviving memory CD4 T cells exhibited intrinsic functional impairments, including reduced production of the proinflammatory cytokines IFNγ, TNFα, and IL-2 upon ex vivo stimulation with the gp_61–80_ peptide. Furthermore, irradiated T cell receptor transgenic memory CD4 T cells exhibited defective secondary expansion upon pathogen re-encounter in unmanipulated hosts. Finally, the irradiated primary memory CD4 T cells exhibited poor protection potential, as they were unable to clear the pathogen upon re-exposure as effectively as their 0 Gy counterpart. In summary, sublethal WBI impairs the kinetics, subset composition, and function of memory CD4 T cells, ultimately compromising their ability to mount effective recall responses to clear the infection.

## Introduction

Radiation was first used as a therapy in 1896, within a few weeks after W.C. Roentgen discovered x-rays, to treat a patient with postoperative recurrent breast cancer.^1^^,^^2^ After a century of technological advances, radiotherapy (RT) has become an integral component of cancer therapy, alongside surgery, chemotherapy, and immunotherapy. RT is now the standard of care, along with chemo/immunotherapy, for more than 60% of cancer types.^3^ Unlike targeted fractionated doses of irradiation used to treat solid tumors, hematologic malignancies require patients to undergo whole-body irradiation (WBI) as a conditioning regimen before bone marrow transplantation. While significant improvements in RT delivery systems have been made to minimize the effects of irradiation on healthy immune and nonimmune cells,^3–5^ RT-mediated tissue toxicity and immune modulation remain prevalent among cancer survivors.^6^^,^^7^ Thus, there is an unmet need to understand the direct effects of radiation exposure on immune cells to better adapt RT approaches to protect hosts from infections following RT.

Innate and adaptive immune cells play vital roles at different stages following an infection to clear the pathogen and hence protect the host. While immediate protection against a new pathogen can be provided by innate immune cells, long-term protection against repeated exposure is provided by adaptive immune cells. The effects of radiation exposure on various immune cells, as studied in mouse models, have provided insights into their radiosensitivity.^8^ Previous studies in humans and mouse models have shown that radiation dose-dependently affects the maintenance of total CD4 and CD8 T cells.^6^^,^^9–11^ While some studies have highlighted the effects of radiation on naïve and memory T cell subsets,^10–13^ these models lacked trackable pathogen-specific T cells to effectively address the direct effects of irradiation on memory T cells. Recent studies from our group have highlighted that targeted irradiation and WBI impair the maintenance and function of tissue-resident and circulating (primary and quaternary) memory CD8 T cells, respectively.^14–16^ However, the impact of irradiation on memory CD4 T cells remains unexplored.

In humans, functional pathogen-specific memory CD4 T cells were identified 75 yr after smallpox vaccination,^17^ 34 yr after measles vaccination,^18^ 21 yr after MMR (measles, mumps, and rubella) vaccination,^19^ and 11 yr after severe SARS-CoV-1 infection.^20^ Several studies in mice have demonstrated that functional CD4 T cell responses are crucial for establishing long-lasting protective CD8 T cell and B cell responses against pathogens.^21–27^ While CD8 T cells primarily protect against intracellular pathogens, CD4 T cells also protect against extracellular bacterial, parasitic, and fungal pathogens.^28–30^ All this leads to an abundance of memory CD4 T cells in healthy humans, which are critical for long-lasting protection. The direct effect of radiation exposure on these pathogen-specific memory CD4 T cells remains elusive. Therefore, it is critical to understand how WBI affects pre-existing memory CD4 T cells. In this study, we use mouse models of infection to demonstrate that irradiation impairs the maintenance and function of memory CD4 T cells.

## Methods

### Mice

Thy1.2 C57Bl/6 mice were purchased from Charles River, and Thy1.1 SMARTA T cell receptor transgenic (TCR-tg) mice were a gift of Noah Butler. Both mouse strains were bred in-house and maintained at the University of Iowa Animal Care Facility at the appropriate biosafety level. Six- to 10-wk-old mice were used for all experiments. Experimental procedures using mice were approved by the University of Iowa Animal Care and Use Committee under protocol number #2121915.

### Adoptive transfer

Thy1.1 SMARTA TCR-tg CD4+ T cells were isolated from the spleens of naïve female donor mice. Spleens were dissociated through a 70 mm filter. Red blood cells were lysed using ACK lysis buffer (in-house). For identification of SMARTA memory, single-cell suspensions were stained with anti-Thy1.1-APC in phosphate-buffered saline (PBS) with 5% fetal calf serum. Frequencies of TCR-tg cells were determined by flow cytometry to estimate cell numbers prior to transfer. A total of 20,000 naïve or 10,000 TCR-tg primary memory CD4 T cells were adoptively transferred via retro-orbital (RO) injection in a total volume of 100 µL into recipient mice 1 d before infection with lymphocytic choriomeningitis virus–Armstrong (LCMV-Arm). For adoptive transfer experiments involving primary memory CD4 T cells, splenocytes from 3 to 5 mice were pooled (within 0 Gy and 5 Gy groups) before transferring into a new host.

### Irradiation

WBI was performed using a 225 kVp rotating x-ray tube (Small Animal Radiation Research platform; Xstrahl) in the radiation cabinet available at the Radiation Core at the University of Iowa. The cabinet delivers a precalibrated dose per minute, and the Core uses the MuriPlan Software (Xstrahl) to calculate the exposure duration required to achieve the desired overall dose. In all experiments, mice were subjected to WBI doses of 1 Gy, 5 Gy, or 10 Gy. It takes ∼8 min of precalibrated continuous exposure to achieve a WBI dose of 5 Gy. So, mice were placed in mouse pie cages and exposed to a duration specific to the WBI dose. Mock-treated (0 Gy) mice were kept in the mouse pie cages inside the cabinet without radiation exposure for the same duration as the highest-dose group (5 Gy or 10 Gy) in each experiment.

### Infections

Mice were infected with 2 × 10^5^ plaque-forming units (PFU) of LCMV-Arm (intraperitoneally [i.p.]) for all experiments.

The generation of specific pathogen–experienced mice was achieved as previously described,^31^ through sequential infection of C57BL/6 specific pathogen–free (SPF) mice, spaced 5 d apart, in the following order: 10^3^ PFU of IAV-PR8 (influenza A virus/Puerto Rico/8/34) intranasally, 10^5^ PFU of murine cytomegalovirus strain Smith or K181 (i.p.), 10^6^ PFU of vaccinia virus (i.p.), 10^7^ colony-forming units (CFU) of attenuated *Listeria monocytogenes* (a.LM) strain DPL19412 (RO, intravenous), and 2 × 10^5^ PFU LCMV-Arm (i.p.).^31^^,^^32^ For secondary challenge experiments, mice were infected with 10^6^ CFU of *actA+InlB*–deficient, gp33+gp61–expressing a.LM (RO, intravenous) or LCMV-Arm (i.p.).

### Tissue processing and cell isolation for flow cytometry

Peripheral blood was collected through the retroorbital sinus. Single-cell suspensions from the spleen, kidneys, and lymph nodes were generated by passing the tissue homogenate through a 70 µm cell strainer without enzymatic digestion. Kidney cells were subsequently run on a 35% Percoll gradient. For bone marrow, both femurs were harvested and crushed with a mortar and pestle, and the tissue homogenate was then passed through a 70 µm cell strainer without enzymatic digestion. ACK lysis buffer was used to lyse red blood cells from peripheral blood, spleen, kidney, and bone marrow samples.

### Cell staining and flow cytometry

To assess antigen-specific CD4 T cell populations, single-cell suspensions were incubated with 50 µL of (1:100) Fc block at 4 °C for 15 min, followed by the addition of 50 µL of PE-conjugated gp_66–77_: I-A^b^ (so that the final concentration is 8 µg/mL in 100 µL; courtesy of National Institutes of Health Tetramer Core Facility) and were incubated at 37 °C for 30 min. Otherwise, the cells were washed and then subjected to surface staining with a cocktail of fluorescently labeled monoclonal antibodies with a 1:100 Fc block at 4 °C for 20 to 30 min and were fixed with Cytofix (BD Biosciences) for 10 min. To label transcription factors, the FoxP3 transcription factor staining kit (Thermo Fisher Scientific) was used, and Bcl-6 was stained at 25 °C for 45 min. To measure cytokine production, 10^6^ splenocytes were plated in the absence or presence of 0.05 or 0.5 µg/mL gp_61–80_ peptide with brefeldin A (BD Biosciences) for 5 h at 37 °C. Cells were washed, stained for surface markers, and then permeabilized using Cytofix/Cytoperm (BD Biosciences) for 15 min. After washing, they were incubated with cytokine-specific monoclonal antibody in PERM wash at 4 °C for 30 to 60 min. For experiments involving CFSE labeling, 10 million splenocytes from each 0 Gy mouse were collected in 15 mL conical tubes and labeled with 1 µM CFSE in PBS for 30 min at 4 °C. Cells were then washed twice with RP10 [RPMI + Supplement + 10% fetal calf serum]. Splenocytes from 5 Gy mice underwent the same steps without CFSE labeling. Both samples were mixed in the end and plated for stimulation with the gp_61–80_ peptide as described previously. Data were acquired using Cytek Aurora (Cytek Biosciences) and analyzed using FlowJo software v.10 (TreeStar). See supplementary table for a complete list of antibodies, clones, and dilutions.

### Antibody depletion

Mice were treated i.p. with 200 μg of depleting anti-CD8α (2.43; BioXCell; BE0061) on 20 and 23 d post-WBI (dpWBI). CD8 T cell depletion was confirmed using flow cytometry on 27 dpWBI by labeling peripheral blood leukocytes (PBLs) from depleted and nondepleted mice using anti-CD8α-PE-Cy7 (53-6.7; BioLegend; 100722) as described previously.

### Bacterial burden enumeration

For enumeration of a.LM, spleen, and liver (were weighed) and collected in 3 mL of sterile PBS to be homogenized. The spleen and liver were homogenized for 30 and 40 s, respectively, at 23,000 rpm using an ULTRA-TURRAX T25 tissue homogenizer. The homogenizer was washed with 70% ethanol between groups, followed by additional washes with PBS and ultrapure water (Type 1 grade) for 30 to 60 s per mouse. The CFU/gram of tissue was determined by plating 10-fold serial dilutions on Tryptic Soy Broth/streptomycin plates. Colony counts were determined after overnight plate incubation at 37 °C.

### Quantification and statistical analysis

All statistical analyses were performed using GraphPad Prism (v9.0–10.4; GraphPad Software). Fold changes were calculated by dividing the means of the groups specified. When indicated, 2-tailed unpaired Student’s *t* tests were performed when comparing two independent groups, 1-way analysis of variance with Tukey’s multiple comparisons test when comparing more than two groups, and 2-way analysis of variance with Sidak’s or Dunnett’s multiple comparisons test when comparing 2 or more groups, respectively, for more than 1 variable. *P* values are indicated in individual figures or figure legends or are otherwise summarized as *P < 0.05, **P < 0.01, ***P < 0.001, ****P < 0.0001.

## Results

### Memory CD4 T cells are susceptible to WBI-induced cell death across different tissues early after radiation exposure

**Figure 1:**
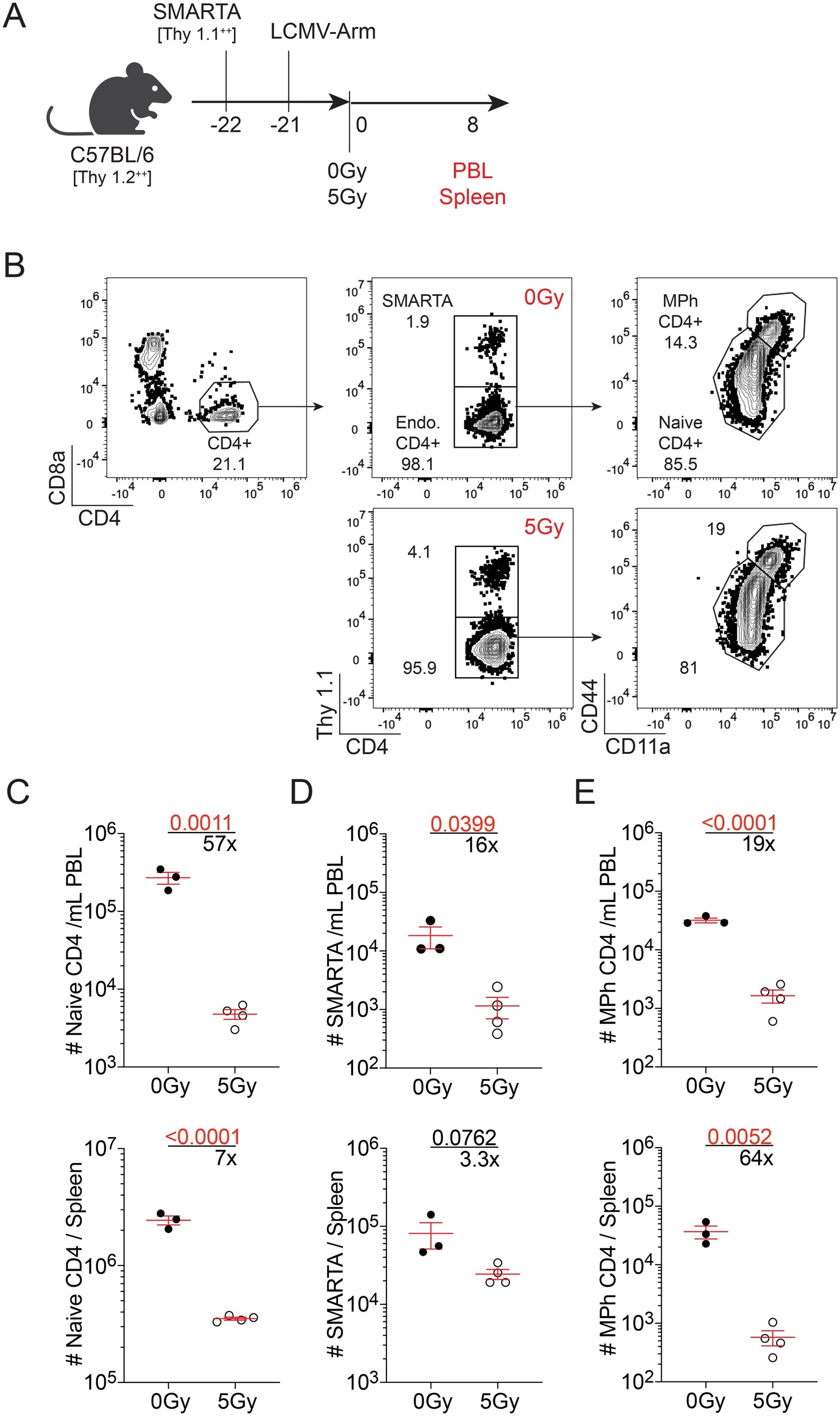
Memory CD4 T cells are susceptible to WBI early after radiation exposure. (A) Experimental design: naïve TCR-tg SMARTA CD4 T cells were adoptively transferred into congenically distinct C57BL/6 mice, followed by LCMV-Arm infection the next day. Three weeks after infection, mice were subjected to 5 Gy or mock (0 Gy) WBI, and the tissues were harvested 8 dpWBI. (B) Representative gating strategy used to identify endogenous naïve (Thy1.1^−^CD44^−^CD11a^lo^), TCR-tg memory (Thy1.1^+^), and MPh (Thy1.1^−^CD44^+^CD11a^hi^) CD4 T cells. The number of naïve (C), TCR-tg memory (D), and MPh (E) CD4 T cells in PBLs (top) and spleen (spleen) are shown. Data are representative of 3 independent experiments with 3 to 4 mice per group. In panels C, D, and E, the fold change between the groups is highlighted. *P* values in panels C, D, and E were derived from 2-tailed unpaired Student’s *t* tests. All graphs show the mean ± SEM. (A) The graphical illustration was created with BioRender.com.

Immune cells exhibit differential susceptibility to radiation-induced death; notably, CD4 T cells are less radiosensitive than CD8 T cells.^9–11^ However, within the CD4 T cell compartment, subsets differ in radiosensitivity. To determine the early effects of sublethal WBI on memory CD4 T cells, we first generated CD4 memory mice. To that end, we adoptively transferred 20,000 congenically distinct (Thy1.1^+/+^) SMARTA TCR-tg naïve CD4 T cells into unmanipulated C57BL/6 mice (Thy1.2^+/+^) and infected them with LCMV-Arm (Fig. 1A). These SMARTA CD4 T cells carry transgenic TCR specific to the gp_61–80_ epitope of LCMV-Arm, which undergo effector expansion followed by contraction to form early memory CD4 T cells in 3 wk after infection.^33–36^ Moving forward, unless specified otherwise, SMARTA chimeric mice challenged with LCMV-Arm were subjected to WBI at 3 wk postinfection. Therefore, at this time point, we subjected these mice to 0 Gy (mock irradiation) or 5 Gy WBI and characterized endogenous (Thy1.1^−^) naïve (CD44^−^CD11a^lo^), memory phenotype (MPh) (CD44^+^CD11a^hi^), and TCR-Tg (Thy1.1^+^) bona fide memory CD4 T cells across different tissues after 8 dpWBI (Fig. 1B).

At 8 dpWBI, in the PBLs of 5 Gy mice, naïve CD4 T cell numbers declined 57-fold (Fig. 1C), whereas virus-specific TCR-tg memory CD4 T cells declined 16-fold (Fig. 1D), compared with the 0 Gy control mice. In the PBLs of 5 Gy mice, the endogenous MP CD4 T cells showed a 19-fold decline compared with the 0 Gy control mice (Fig. 1E). These MPh CD4 T cells comprise both endogenous virus-specific, bona fide memory and antigen-inexperienced virtual memory–like CD4 T cells. This pattern of severe fold loss in naïve CD4 T cells, compared with the fold loss in TCR-tg memory CD4 T cells at 8dpWBI, was also observed in the spleen, mesenteric lymph node, bone marrow, and kidneys (Fig. 1; Fig. S1). Overall, these data suggest that early after irradiation (8 dpWBI), naïve CD4 T cells sustain a more severe fold loss than TCR-tg memory CD4 T cells across different tissues.

### Memory CD4 T cells sustain permanent numerical loss following WBI

**Figure 2:**
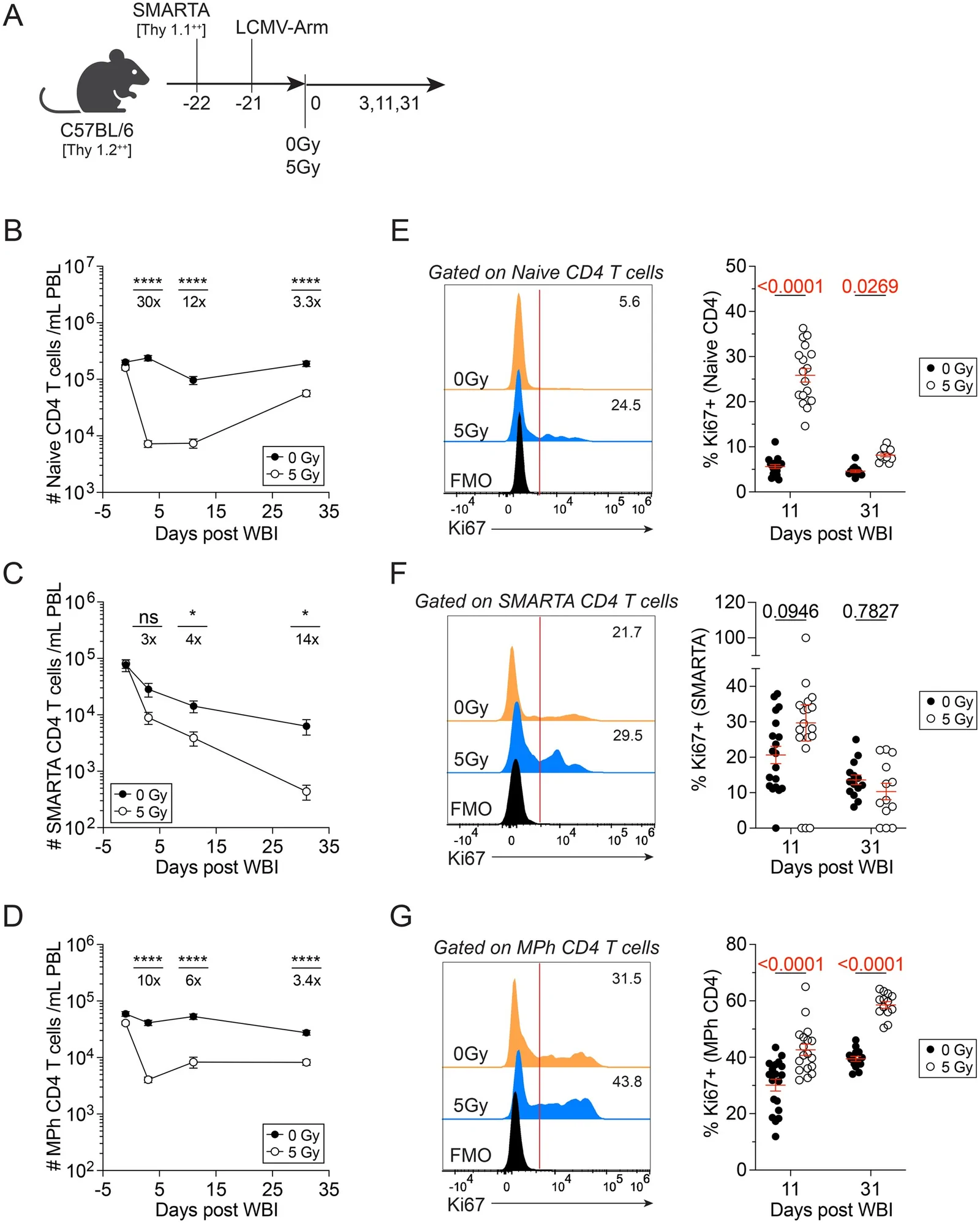
Memory CD4 T cells sustain permanent numerical loss following WBI. (A) Experimental design: naïve TCR-tg SMARTA CD4 T cells were adoptively transferred into congenically distinct C57BL/6 mice, followed by LCMV-Arm infection the next day. Three weeks after infection, mice were subjected to 5 Gy or mock (0 Gy) WBI, and their blood was collected at 3, 11, and 31 dpWBI. The kinetics of the number of naïve (B), TCR-tg memory (C), and MPh (D) CD4 T cells per mL of PBLs. The histogram (left) and the frequency (right) of Ki-67–expressing naïve (E), TCR-tg memory (F), and MPh (G) CD4 T cells at 11 and 31 dpWBI. Data are representative of 3 independent experiments with at least 13 mice per group. In panels B, C, and D, the fold change between the groups at the specified dpWBI is highlighted. Statistical significance derived from 2-way analysis of variance with Sidak’s multiple comparisons test: *P ≤ 0.05 and ****P ≤ 0.0001. *P* values in panels E, F, and G were derived from 2-way analysis of variance with Sidak’s multiple comparisons test. All graphs show the mean ± SEM. (A) The graphical illustration was created with BioRender.com. ns, not significant.

To determine the long-term effects of WBI on circulating memory CD4 T cell numbers, we generated the SMARTA chimeric mice and subjected them to 0 Gy or 5 Gy WBI. We then assessed the number of naïve, TCR-tg memory, and MPh CD4 T cells in the PBLs up to 31 dpWBI (Fig. 2A). After 3 dpWBI, the number of naïve, TCR-tg memory, and MPh CD4 T cells declined in numbers compared with 0 Gy mice (Fig. 2B–D). While naïve CD4 T cells declined up to 10 dpWBI, they increased in number around 31 dpWBI (Fig 2B). In contrast, TCR-tg memory CD4 T cells sustained further decline (Fig. 2C), increasing the fold loss from 4-fold at 10 dpWBI to 14-fold at 31 dpWBI. The latter results suggested that WBI causes a sustained decline in bona fide memory CD4 T cells over time. As for the endogenous MPh CD4 T cells, the numbers declined early after WBI and then maintained at a similar level up to 31 dpWBI, yet remained 3.4-fold lower than 0 Gy mice (Fig. 2D). This may be attributable to contributions from both antigen-inexperienced, virtual memory–like CD4 T cells and virus-specific, polyclonal memory CD4 T cells to the endogenous CD44^+^CD11a^hi^ CD4 T cell compartment. Unlike CD8 T cells,^37–40^ antigen-inexperienced virtual memory cells are poorly defined among CD4 T cells. While recent studies have described these as “MPh” CD4 T cells in noninfected SPF and germ-free mice,^41–43^ a marker-based approach to distinguish these CD4 T cells from pathogen-specific bona fide memory CD4 T cells among infected hosts is currently lacking.

Studies over the past two decades have shown that naïve CD4 T cells in a lymphopenic environment undergo two types of proliferation to reconstitute the CD4 T cell compartment.^44–47^ The IL-7–dependent homeostatic proliferation (HP) maintains the naïve (CD44^−^) phenotype of CD4 T cells, whereas spontaneous rapid proliferation leads to differentiated effector-like (CD44^+^) naïve CD4 T cells. In particular, WBI-mediated lymphopenia induces IL-7–dependent HP in both CD44^+^ and CD44^−^ CD4 T cells.^13^ To determine whether naïve and MPh CD4 T cells in irradiated hosts undergo increased proliferation after 11 dpWBI to achieve numerical recovery and maintenance, respectively, we measured the frequency of Ki-67 expression in these cells, a nuclear protein expressed by actively proliferating cells. It was evident that both (CD44^−^) naïve and (CD44^+^) MPh CD4 T cells in 5 Gy mice are actively proliferating compared with the 0 Gy mice from 11 to 31 dpWBI (Fig. 2E, G). This suggests that the reconstitution of these CD4 T cell subsets is aided by active proliferation (Fig. 2B, D, E, and G). While HP is essential, thymic reconstitution has also been shown to play a critical role in the recovery of naïve CD4 and CD8 T cells following WBI.^38^^,^^48^ However, irradiated TCR-Tg memory CD4 T cells express similar levels of Ki-67 as the 0 Gy mice after 11 and 31 dpWBI (Fig. 2F). This suggests that the continued numerical decline in TCR-tg memory CD4 T cells following WBI (Fig. 2C) is potentially due to their unaltered proliferation and due to the inability to generate new SMARTA precursors from the thymus. Overall, this indicates that different subsets of CD4 T cells in peripheral circulation exhibit varying levels of radiosensitivity and recovery kinetics, with the memory CD4 T cells sustaining permanent numerical loss following WBI.

### Both TCR-tg and polyclonal memory CD4 T cells in the spleen exhibit sustained numerical decline following WBI

**Figure 3:**
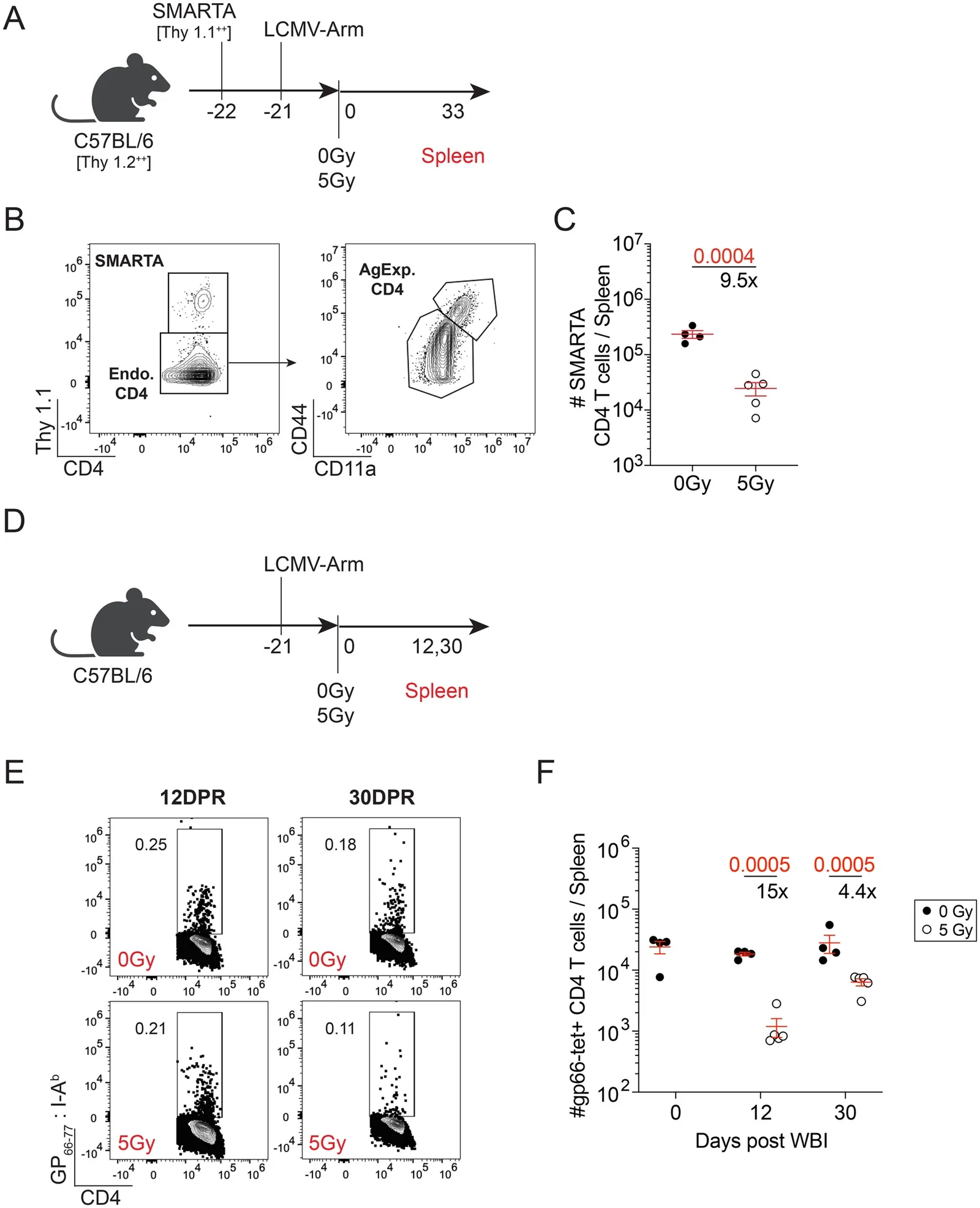
Both TCR-tg and polyclonal memory CD4 T cells in the spleen exhibit sustained numerical decline following WBI. (A) Experimental design: upon generating TCR-tg memory CD4 T cell chimeric mice, they were subjected to 5 Gy or mock (0 Gy) WBI, and the spleen was harvested 33 dpWBI. Representative flow plots (B) and the number (C) of TCR-tg memory CD4 T cells in the spleen, comparing mice described in panel A. (D) Experimental design: upon generating virus-specific polyclonal memory CD4 T cells in mice lacking TCR-tg cells, they were subjected to 5 Gy or mock (0 Gy) WBI, and the spleen was harvested 12 and 33 dpWBI. Representative flow plots (E) and the number (F) of GP_66–77_-tetramer-specific polyclonal memory CD4 T cells in the spleen, comparing mice described in panel D. Data are representative of 2 independent experiments with 4 to 5 mice per group. In panels C and F, the fold change between the groups is highlighted. *P* value in panels C and F were derived from 2-tailed unpaired Student’s *t* test and 2-way analysis of variance with Sidak’s multiple comparisons test, respectively. All graphs show the mean ± SEM. (A, D) The graphical illustrations were created with BioRender.com.

We showed that the TCR-tg memory CD4 T cells in circulation undergo a sustained numerical decline following WBI (Fig. 2C). While the TCR-tg memory CD4 T cells in the spleen, after 8 dpWBI, exhibit a 3.3-fold decline (Fig. 1D), whether this decline continues remains to be tested. To that end, we generated SMARTA chimeric mice and subjected them to 0 Gy or 5 Gy WBI (Fig. 3A). After 33 dpWBI—or 54 d post-LCMV-Arm infection–the TCR-tg memory CD4 T cell numbers were significantly lower in the WBI mice (Fig. 3B, C). In fact, the 3.3-fold decrease observed in the number of TCR-tg memory CD4 T cells in the spleen at 8 dpWBI (Fig. 1D) is now increased to a 9.5-fold decline by 33 dpWBI (Fig. 3C). This suggests that the TCR-tg memory CD4 T cells that cannot be regenerated from new precursors sustain permanent numerical loss following WBI. We next set out to test if the polyclonal virus-specific endogenous memory CD4 T cells, which can be regenerated through thymic reconstitution and HP,^48^ also exhibit numerical decline following WBI. To that end, wild-type C57BL/6 mice were infected with LCMV-Arm and subjected to 0 Gy or 5 Gy WBI after 3 wk postinfection (Fig. 3D). After 12 and 30 dpWBI, spleens were harvested from these mice to assess the polyclonal gp_66–77_ tetramer–specific endogenous memory CD4 T cells (Fig. 3E). At both of these time points, the WBI mice exhibit a severe decline in polyclonal memory CD4 T cells compared with their 0 Gy counterparts (Fig. 3F). While the polyclonal gp_66–77_ tetramer–specific endogenous memory CD4 T cells exhibit a 15-fold decline at 12 dpWBI, it changes to 4.4-fold decline by 30 dpWBI. This is potentially driven by the regeneration of polyclonal virus-specific precursors through HP and thymic reconstitution following WBI,^38^^,^^48^ yet the numbers remain lower than those of 0 Gy counterparts. Overall, these data suggest that WBI leads to sustained numerical loss of both TCR-tg and polyclonal memory CD4 T cells.

### WBI transiently alters the composition of the memory CD4 T cell subsets and their phenotype

Unlike CD8 T cells, defining memory CD4 T cell subsets have been challenging, owing to their plasticity, heterogeneity, and functional diversity.^49^^,^^50^ The CD127, KLRG1, and CD62L-based definition of short-lived effector cells, memory precursor effector cells, effector memory (T_EM_), and central memory (T_CM_) T cell subsets, which is well established for memory CD8 T cells, has proven ineffective for defining memory CD4 T cell subsets.^36^^,^^50^ Intracellular pathogens such as LCMV-Arm preferentially generate T helper 1 (Th1) and T follicular helper (Tfh) cells during the peak effector and memory stages.^33–36^^,^^51^ Marshall et al.^36^ showed, for the first time in a mouse model of LCMV infection, that PSGL1 and Ly6C expression can be used to identify Th1 (PSGL1^hi^Ly6C^hi^) and Tfh (PSGL1^lo^Ly6C^lo^) CD4 T cell subsets at effector and memory time points. Several recent studies have identified an additional T_CM_ (PSGL1^hi^Ly6C^lo^) subset that coexists with Th1 and Tfh memory subsets and is transcriptionally distinct.^35^^,^^36^^,^^51^ Kunzli et al.^35^ showed that, upon adoptive transfer of PSGL1^hi^Ly6C^lo^ and PSGL1^hi^Ly6C^hi^ memory CD4 T cells into a new host, followed by secondary challenge, Ly6C^hi^ CD4 T cell donors predominantly remained Ly6C^hi^, while Ly6C^lo^ CD4 T cell donors generated both Ly6C^hi^ and Ly6C^lo^ memory CD4 T cells. This suggested that the PSGL1^hi^Ly6C^lo^ and PSGL1^hi^Ly6C^hi^ memory CD4 T cells are T_CM_ and Th1 subsets, respectively. Whereas PSGL1^lo^Ly6C^lo^ Tfh cells can generate all 3 subsets during recall responses in a new host.^35^ Such studies have strengthened the use of the PSGL1- and Ly6C-based approach for defining memory CD4 T cells.^35^^,^^51^

To test if WBI affects these different memory CD4 T cell subsets, we generated SMARTA chimeric mice and subjected them to 0 Gy or 5 Gy WBI (Fig. S2A). After 8 dpWBI—or 29 d post-LCMV-Arm infection—the TCR-tg memory CD4 T cells in both 0 Gy and 5 Gy mice were primarily composed of Ly6C^hi^ Th1 cells in circulation (PBLs) and secondary lymphoid organs (SLOs) (spleen and mesenteric lymph node) (Fig. S2B–E). In contrast, the T_CM_ and Tfh subsets of TCR-tg memory CD4 T cells were predominantly in the SLOs. Interestingly, TCR-tg memory CD4 T cells from 5 Gy mice showed an increased frequency of Ly6C^hi^ Th1 cells but a decreased frequency of T_CM_ cells in the SLOs, compared with 0 Gy mice (Fig. S2D, E). These data suggest that early after radiation exposure, memory CD4 T cell subsets in WBI mice skew toward the Th1 subset and away from the T_CM_ subset, compared with 0 Gy mice. This was also true among endogenous MPh CD4 T cells (CD44^+^CD11a^hi^), in which WBI mice showed an increased frequency of Th1 subsets and a decreased frequency of T_CM_ and Tfh subsets compared with 0 Gy mice in the SLOs. To test whether the imbalance in subset composition observed in TCR-tg memory CD4 T cells of irradiated mice persisted long-term, we assessed the same, after 33 dpWBI from mice described in Figure 3A. At this time point, the frequency of all 3 memory CD4 T cell subsets remained similar between 0 Gy and 5 Gy mice (Fig. S3A–C), suggesting that the alterations to the memory CD4 T cell subset composition following WBI are transient. SMARTA TCR-tg CD4 T cells are known to favor Th1 subset differentiation in response to LCMV-Arm infection in mice.^52^ However, endogenous polyclonal memory CD4 T cells are predominantly T_CM_ at the memory time point (Fig. S3F, H). At 12 dpWBI, the polyclonal memory CD4 T cells from 5 Gy mice showed an increased frequency of T_CM_ cells but a decreased frequency of Tfh cells in the spleen, compared with 0 Gy mice (Fig. S3F). Unlike TCR-tg memory, the Th1 subset composition of polyclonal memory CD4 T cells remained similar between WBI and 0 Gy mice. Similar to TCR-tg memory, the alteration in subset composition in polyclonal memory CD4 T cells was also transient, as Th1 and T_CM_ levels were comparable between WBI and 0 Gy mice, with the decline in Tfh still persisting but resolving after 30 dpWBI (Fig. S3H). Together, these findings show that both the TCR-tg and polyclonal memory CD4 T cells undergo transient alterations in subset composition that resolve over time.

Next, we determined the frequency of cells expressing CXCR3, CD62L, Bcl-6, and PD-1 within these TCR-tg memory CD4 T cell subsets, together with their per-cell expression (geometric mean fluorescence intensity), at 33 dpWBI in the mice described in Figure S3A (Fig. S4). Following WBI, the frequency of CXCR3⁺ cells was unchanged in the Th1 and T_CM_ subsets, whereas their per-cell CXCR3 expression declined markedly relative to 0 Gy control mice, indicating a per-cell downregulation rather than loss of the CXCR3⁺ population (Fig. S4A). For CD62L, both the frequency and the per-cell expression of CD62L among Th1 cells decreased among WBI mice relative to 0 Gy control mice, with no change in the T_CM_ or Tfh subsets (Fig. S4B). Both the frequency and per-cell level expression of Bcl-6 remained unchanged across all 3 subsets (Fig. S2C). Interestingly, both the frequency and per-cell expression of PD-1 were higher in the Th1 and T_CM_ subsets of WBI mice relative to 0 Gy control mice, while the same in Tfh cells remain unchanged (Fig. S4D). Together, these data show that WBI most robustly increases PD-1 expression in the Th1 and T_CM_ subsets of TCR-tg memory CD4 T cells, alongside a selective per-cell reduction in CXCR3.

**Figure 4:**
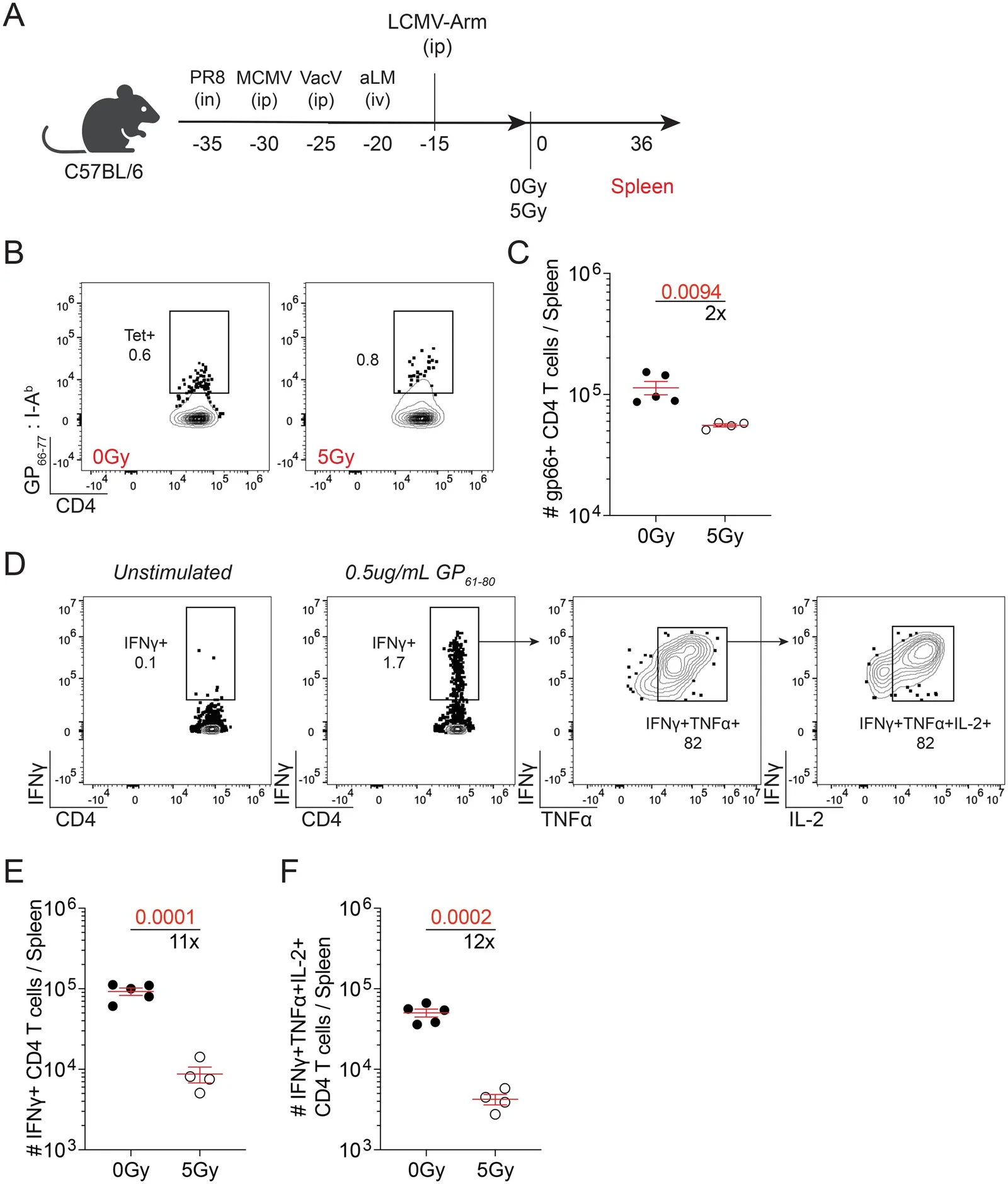
Pathogen-specific endogenous memory CD4 T cells that produce proinflammatory cytokines are limited in irradiated physiologic pathogen–experienced mice. (A) Experimental design: SPF C57BL/6 mice were exposed to 5 peripheral pathogens spaced 5 d apart to generate specific pathogen–experienced (SPExp) mice. The peripheral pathogens were infected in the order: IAV-PR8, murine cytomegalovirus (MCMV), vaccinia virus (VacV), a.LM, and LCMV-Arm. At 15 d post–LCMV infection, mice were subjected to 0 Gy or 5 Gy WBI, and their spleens were harvested 36 dpWBI. Representative flow plots (B) and the number (C) of gp_66–77_ tetramer–specific CD4 T cells from the spleen of the mice described in panel A. (D) Representative flow plots (0 Gy) and the gating strategy used to find IFNγ, TNFα, and IL-2 expression among endogenous MPh CD4 T cells upon 0.5 µg/mL gp_61–80_ peptide stimulation. The number of endogenous MPh CD4 T cells producing IFNγ (E), and all 3 cytokines, namely IFNγ, TNFα, and IL-2 (F), in the spleen of mice described in panel A. Data are representative of 2 independent experiments with 4 to 5 mice per group. In panels C, E, and F, the fold change between the groups is highlighted. *P* values were derived from 2-tailed unpaired Student’s *t* tests. All graphs show the mean ± SEM. (A) The graphical illustration was created with BioRender.com.

### Functional pathogen-specific endogenous memory CD4 T cells are limited in physiologic pathogen–experienced hosts following WBI

Our data demonstrate that the number of TCR-tg memory CD4 T cells following WBI continually declines over time, both in the periphery (Fig. 2F) and in the spleen (Figs. 1E and 3C). WBI also led to a 4.4-fold decline in the number of polyclonal memory CD4 T cells in the spleen compared with 0 Gy mice after 30 dpWBI (Fig. 3F). While these mice were maintained in a controlled, SPF environment, humans experience exposure to diverse pathogens prior to radiation exposure, which can regulate the memory CD4 T cell response. To test the effect of WBI on polyclonal memory CD4 T cells in a mouse model with a history of physiologic microbial exposure, we used the previously well-established mouse model of specific pathogen exposure.^31^^,^^32^ In this model, mice were sequentially infected with IAV-PR8, murine cytomegalovirus, vaccinia virus, a.LM, and LCMV-Arm, with a 5-d interval between infections. After 15 d post-LCMV challenge, mice were subjected to either 0 Gy or 5 Gy WBI (Fig. 4A). After 36 dpWBI, the LCMV epitope gp_66–77_ tetramer–specific endogenous memory CD4 T cells were 2× lower in the WBI mice compared with 0 Gy mice (Fig. 4B, C). To test if these endogenous memory CD4 T cells can produce proinflammatory cytokines upon cognate-antigen recognition, the splenocytes were stimulated with a physiologically relevant dose of the gp_61–80_ peptide. Upon stimulation with gp_61–80_ peptide, the number of polyclonal memory CD4 T cells capable of producing IFNγ alone or in combination with TNFα and IL-2 was significantly lower in the WBI mice compared with the 0 Gy mice (Fig. 4D–F). Overall, this suggests that WBI severely impairs the number and the cytokine-producing function of pathogen-specific endogenous memory CD4 T cells even in a host with a history of physiologic microbial exposure.

### WBI causes per-cell level cytokine dysfunction among memory CD4 T cells in a T cell–intrinsic manner

**Figure 5:**
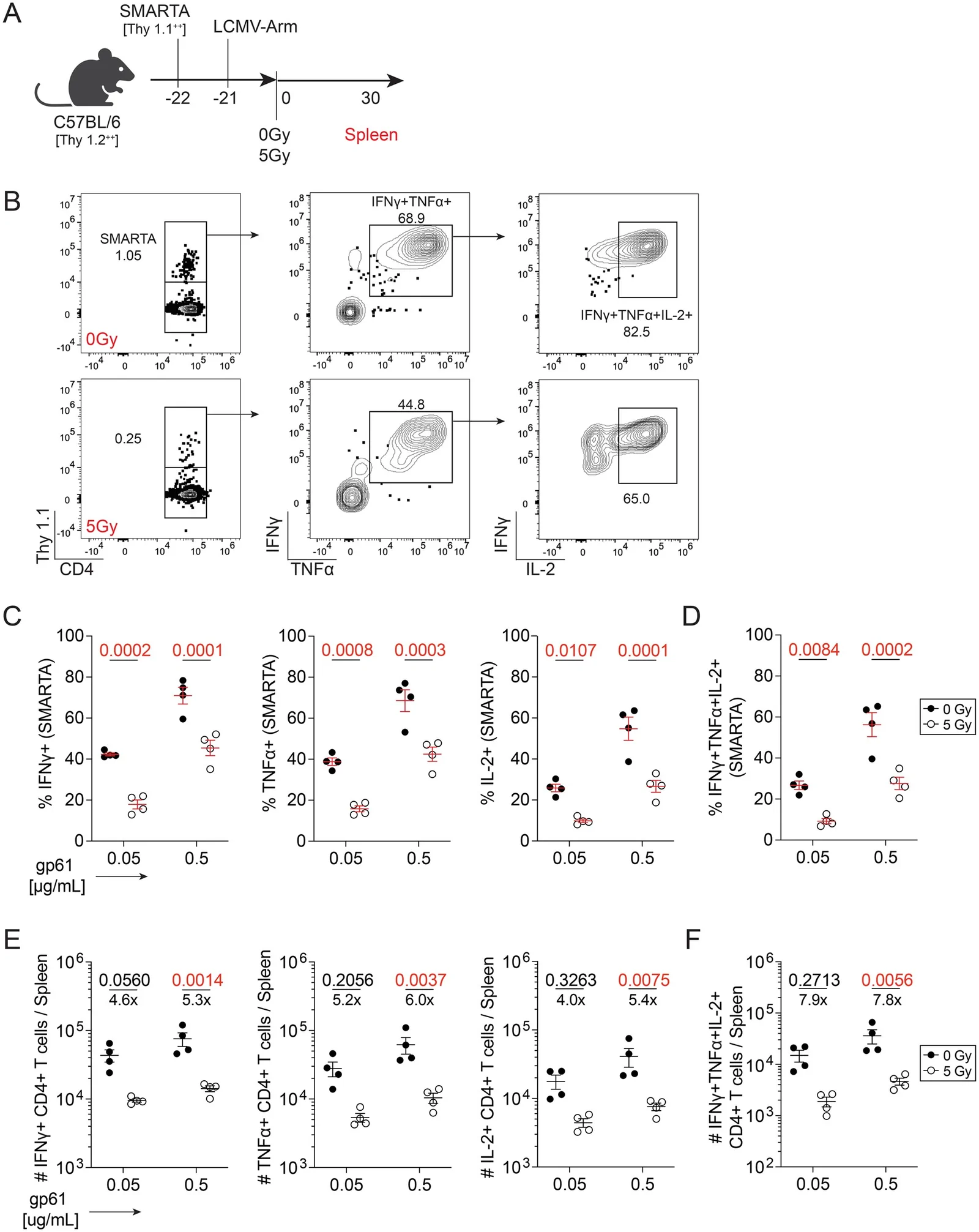
WBI causes per-cell-level cytokine dysfunction in memory CD4 T cells. (A) Experimental design: upon generating TCR-tg memory CD4 T cell chimeric mice, they were subjected to 5 Gy or mock (0 Gy) WBI, and the spleen was harvested 30 dpWBI. (B) Representative flow plots and the gating strategy used to find IFNγ, TNFα, and IL-2 expression among TCR-tg memory CD4 T cells upon 0.5 µg/mL gp_61–80_ peptide stimulation. (C) The frequency of TCR-tg memory CD4 T cells producing single cytokines, namely IFNγ, TNFα, and IL-2. (D) The frequency of TCR-tg memory CD4 T cells simultaneously producing IFNγ, TNFα, and IL-2. (E) The number of MPh CD4 T cells producing single cytokines, namely IFNγ, TNFα, and IL-2. (F) The number of MPh CD4 T cells simultaneously producing IFNγ, TNFα, and IL-2. Data are representative of 3 independent experiments with 4 mice per group. *P* values in panels C, D, E, and F were derived from 2-way analysis of variance with Sidak’s multiple comparisons test. All graphs show the mean ± SEM. (A) The graphical illustration was created with BioRender.com.

While WBI is shown to reduce the number of pathogen-specific memory CD4 T cells by 2× (Fig. 4C), the number of memory CD4 T cells capable of producing IFNγ in response to ex vivo peptide stimulation is 11× lower than in 0 Gy mice (Fig. 4E). This suggests that apart from the numerical deficit, WBI also affects the cytokine response potential of memory CD4 T cells at the per-cell level. The TCR-tg CD4 T cell adoptive transfer system is better suited to address this question, as it allows us to measure the frequency of cytokine producers among the defined TCR-tg memory CD4 T cells. To that end, we adoptively transferred TCR-tg naïve CD4 T cells into congenically distinct recipient mice and infected them with LCMV-Arm. Three weeks postinfection, mice were subjected to either 0 Gy or 5 Gy WBI (Fig. 5A). After 30 dpWBI, splenocytes were stimulated with two different doses of gp_61–80_ peptide. Gating into TCR-tg memory CD4 T cells, we identified IFNγ, TNFα, and IL-2–producing cells (Fig. 5B). First, memory CD4 T cells from 0 Gy mice showed a gp_61–80_ peptide dose-dependent cytokine response (Fig. 5C). Second, both doses of gp_61–80_ peptide stimulation showed a significant decline in the frequency of irradiated TCR-tg memory CD4 T cells producing IFNγ, TNFα, and IL-2 cytokines (Fig. 5C). While single cytokine producers are important, memory T cells capable of producing multiple proinflammatory cytokines are well established to be beneficial in intracellular infections.^53^^,^^54^ Here, we observe that the frequency of irradiated TCR-tg memory CD4 T cells capable of simultaneously producing IFNγ, TNFα, and IL-2 is significantly lower in 5 Gy mice (Fig. 5B, D). Overall, this suggests a per-cell-level impairment in proinflammatory cytokine production among memory CD4 T cells following WBI. Upon gp_61–80_ peptide stimulation, the number of polyclonal memory CD4 T cells capable of producing single or multiple (IFNγ, TNFα, and IL-2) cytokines was also significantly lower in the WBI mice compared with the 0 Gy mice (Fig. 5E, F). Overall, this points to WBI-mediated impairment of the cytokine-response potential of both TCR-tg and polyclonal memory CD4 T cells.

Next, we set out to determine whether the inability of irradiated memory CD4 T cells to mount a robust proinflammatory cytokine response is intrinsic to CD4 T cells or due to extrinsic regulation. To test this, we used a model similar to that described earlier (Fig. S5A) and labeled splenocytes from the 0 Gy mice with CFSE, leaving those from the 5 Gy mice unlabeled. Subsequently, an equal number of splenocytes from 0 Gy and 5 Gy mice was mixed in the same well (Fig. S5B) to normalize cell-extrinsic factors (non–T cells) between groups. These cells were then stimulated with the same two doses of gp_61–80_ peptide, as described previously. Using CFSE labeling, we distinguished total CD4 T cells from 0 Gy and 5 Gy mice, followed by identification of TCR-tg memory CD4 T cells based on Thy1.1 expression (Fig. S5C). As previously shown in Figure 5, a significantly lower frequency of irradiated TCR-tg memory CD4 T cells produced the proinflammatory cytokines IFNγ, TNFα, and IL-2 compared with the mock-irradiated CD4 T cells in the same well (connected on the graph by lines) (Fig. S3D, E). This shows that despite normalizing the ex vivo environment, the memory CD4 T cell dysfunction following WBI could not be remedied. Overall, this suggests that the WBI-mediated per-cell immune dysfunction observed in memory CD4 T cells is intrinsic to these cells.

### WBI impairs the expansion potential of memory CD4 T cells during rechallenge

**Figure 6:**
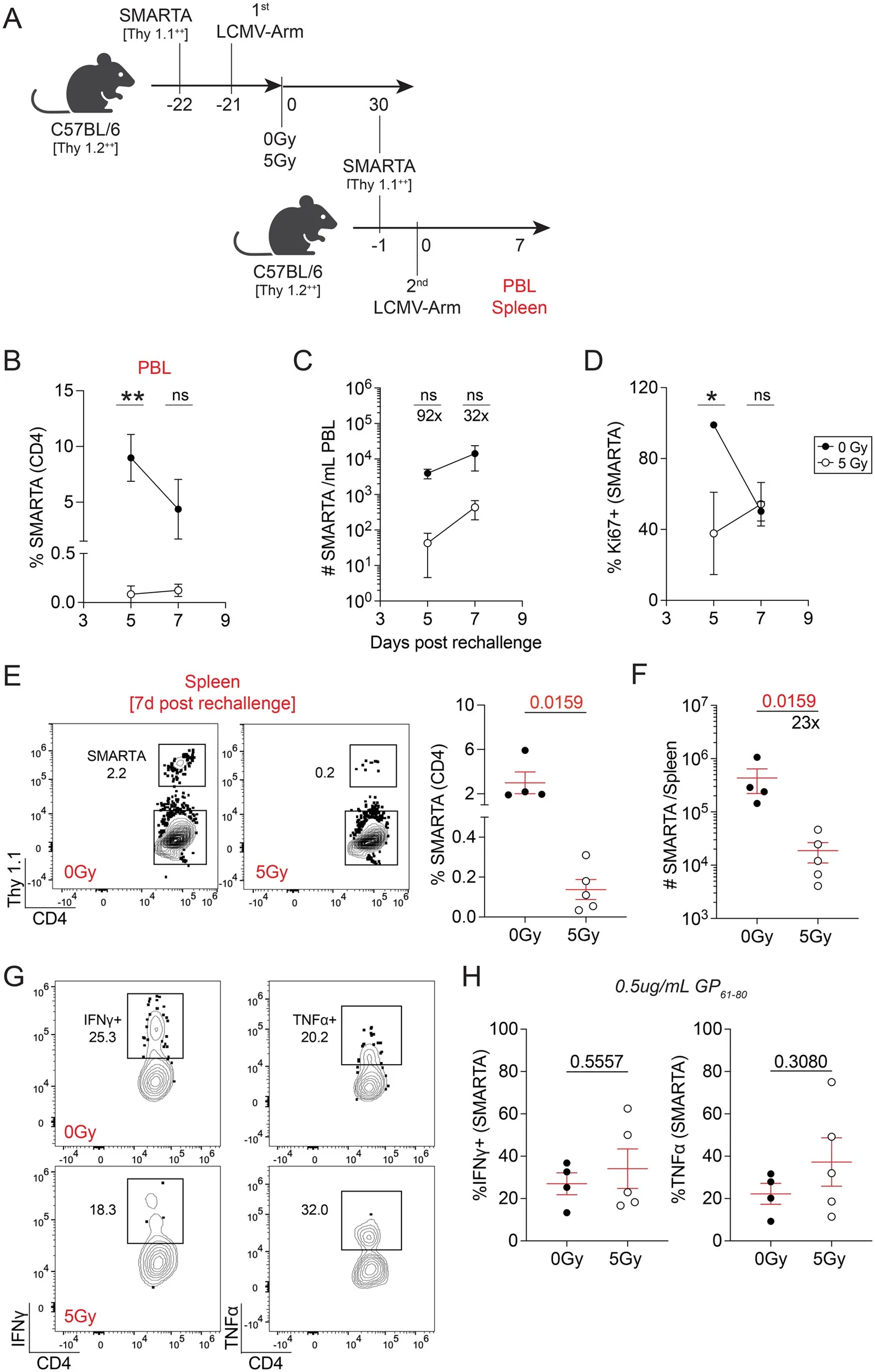
WBI impairs the expansion potential of memory CD4 T cells during rechallenge. (A) Experimental design: SMARTA chimeric mice infected with LCMV-Arm were subjected to 5 Gy or mock (0 Gy) WBI, and the splenocytes were harvested 30 dpWBI. Splenocytes carrying 10,000 TCR-tg primary memory CD4 T cells were transferred into unmanipulated new naïve mice, followed by LCMV-Arm infection the next day. PBLs and spleen were harvested at specified time points. The frequency (B) and the number (C) of TCR-tg memory CD4 T cells in the PBLs of the mice described in panel A. (D) The frequency of TCR-tg memory CD4 T cells expressing Ki-67 at specified time points in the PBLs. Representative flow plots, frequency (E), and the number (F) of TCR-tg memory CD4 T cells in the spleen after 7 d postrechallenge. Representative flow plots (G) and the frequency (H) of IFNγ-producing (left) and TNFα-producing (right) TCR-tg memory T cells upon 0.5 µg/mL gp_61–80_ peptide stimulation in mice described in panel A. Data are representative of 2 independent experiments with 4 to 5 mice per group. *P* values in panels B, C, and D were derived from 2-way analysis of variance with Sidak’s multiple comparisons test: *P ≤ 0.05 and **P ≤ 0.01. *P* values in panels E, F, and H were derived from 2-tailed unpaired Student’s *t* tests. All graphs show the mean ± SEM. Fold change between the groups is highlighted in panels C and F. (A) The graphical illustration was created with BioRender.com. ns, not significant.

Another hallmark of memory CD4 T cells is their capacity to expand numerically upon antigen re-exposure. To address the impact of WBI on recall potential, we used the SMARTA chimeric mouse model of LCMV-Arm infection as previously described, and after 30 dpWBI, splenocytes carrying equal number of TCR-tg primary memory CD4 T cells from 0 Gy and 5 Gy mice were transferred into congenically distinct unmanipulated naïve hosts (Fig. 6A). This generates a mouse model in which the primary memory CD4 T cells from 0 Gy and 5 Gy mice are in a new nonirradiated and uninfected host, thus normalizing all the CD4 T cell–extrinsic factors that can influence their response to a LCMV-Arm infection. Upon infecting these new hosts with LCMV-Arm, the primary memory TCR-Tg CD4 T cells should mount a robust secondary T cell expansion, while the host’s endogenous T cell response will be a primary T cell response.

Five to 7 d after rechallenge, the frequency and number of TCR-tg memory CD4 T cells in PBLs were significantly lower in 5 Gy recipients than in 0 Gy recipients (Fig. 6B, C). Ki-67, a transcription factor expressed at high levels in actively dividing cells, was used to assess differences in cell-cycle potential between 0 Gy and 5 Gy TCR-tg CD4 T cells. After 5 d postrechallenge, the frequency of TCR-tg memory CD4 T cells expressing Ki-67 was higher among 0 Gy recipients compared with 5 Gy recipients, while these levels were similar between the groups by day 7 postrechallenge (Fig. 6D). This suggests that the expansion potential of irradiated memory CD4 T cells to rechallenge is severely impaired in a new unmanipulated host. Additionally, after 7 d postrechallenge, the frequency and number of TCR-tg memory CD4 T cells in the spleen were significantly lower in 5 Gy recipients than in 0 Gy recipients (Fig. 6E, F). To test the cytokine-producing capacity of the recalled SMARTA cell, sp from 7 d postrechallenge were stimulated with the gp_61–80_ peptide (0.5 µg/mL). Similar frequencies of TCR-tg memory CD4 T cells from both 0 Gy and 5 Gy mice produced IFNγ and TNFα (Fig. 6G, H). This suggests that those TCR-Tg memory CD4 T cells from 5 Gy mice that could undergo secondary expansion can match the cytokine response of memory CD4 T cells derived from 0 Gy hosts. Overall, these data indicate that WBI leads to intrinsic impairment of memory CD4 T cell expansion potential following rechallenge in a new host.

### WBI impairs the memory CD4 T cell–mediated pathogen clearance during secondary exposures

**Figure 7:**
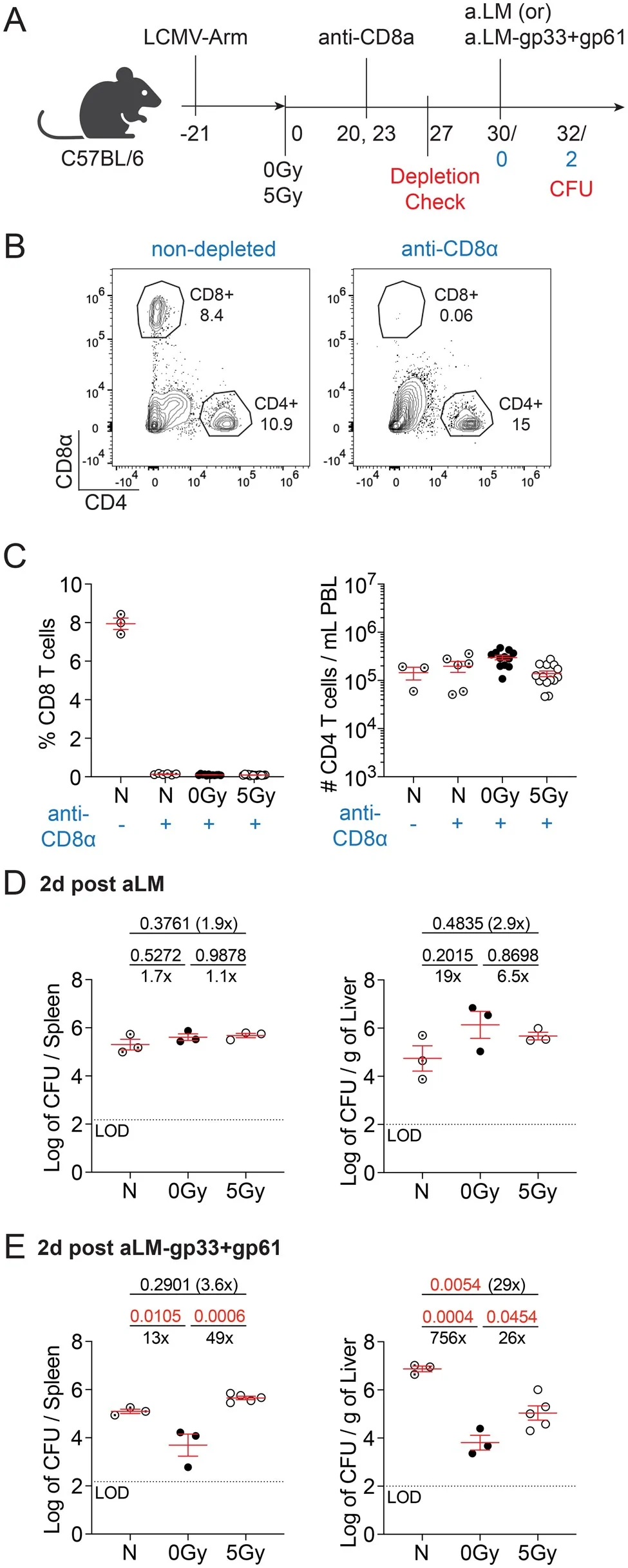
WBI impairs memory CD4 T cell–mediated pathogen clearance during secondary exposures. (A) Experimental design: SPF C57BL6 mice were infected with LCMV-Arm, and after 3 wk were subjected to 5 Gy or mock (0 Gy) WBI. All mice in both groups, as well as uninfected naïve mice, received anti-CD8α at 20 and 23 dpWBI. These mice were then infected with either a.LM or a.LM-gp33+gp61, and their spleen and liver were harvested after 2 d. Representative flow plots (B), the frequency of CD8 T cells (C, left), and the number of CD4 T cells (C, right) in the blood are shown to confirm CD8 T cell depletion before a.LM infection. (D) At 2 d after a.LM infection, the log of CFU in the spleen (left) and liver (right) of the mice is described in panel A. (E) After 2 d from a.LM-gp33+gp61 infection, the log of CFU in the spleen (left) and the liver (right) of mice is described in panel A. Data are a representative of a single experiment with at least 3 mice per group. *P* values in panels D and E were derived from 1-way analysis of variance with Sidak’s multiple comparisons test. All graphs show the mean ± SEM. Fold change between the groups is highlighted. (A) The graphical illustration was created with BioRender.com.

So far, our data have comprehensively demonstrated that WBI leads to a numerical decline in memory CD4 T cells and cytokine dysfunction and subsequently limits expansion upon rechallenge. In an unmanipulated host, the memory functions of CD4 T cells work together to clear the pathogen during a secondary challenge. However, this raises questions about the pathogen-clearing capacity of irradiated memory CD4 T cells during secondary exposure to the pathogen. To directly address this question, we infected mice with LCMV-Arm and, after 3 wk, subjected them to 5 Gy or mock WBI. After 20 dpWBI, all mice in the study were subjected to CD8 T cell depletion before being infected with either a.LM or a.LM expressing gp33 and gp61 peptide (a.LM-gp33+gp61) (Fig. 7A). Age-matched, uninfected (No LCMV-Arm) and nonirradiated naïve mice were also subjected to CD8 depletion. After 27 dpWBI (before a.LM challenge), PBLs from all the groups showed successful CD8 T cell depletion, while nondepleted naïve mice retained their CD8 T cells (Fig. 7B, C). CD8 T cell depletion did not influence the number of CD4 T cells in the PBLs, as they were similar across all the groups (Fig. 7C). Thus, after 30 dpWBI, unlike the 0 Gy and 5 Gy mice, naïve mice should not have LCMV-specific CD4 T cells, while all 3 groups should be devoid of CD8 T cells.

We observed in the spleen and liver of naïve, 0 Gy, and 5 Gy mice that the pathogen burden is similar at 2 d postinfection with a.LM that lack known LCMV epitopes (Fig. 7D). However, upon challenging these mice with a.LM-gp33+gp61, the pathogen burden in the spleen was significantly declined in the 0 Gy mice compared with the naïve mice (Fig. 7E), suggesting that the pre-existing functional memory CD4 T cells are critical for clearing the pathogen in the absence of CD8 T cells. Interestingly, the pathogen burden in the spleen of 5 Gy mice was similar to that of naïve mice and significantly higher than that of the 0 Gy mice (Fig. 7E). In the liver, the pathogen burden was lower among both 0 Gy and 5 Gy mice compared with the naïve mice; however, the pathogen burden among 5 Gy mice was still significantly higher than the 0 Gy mice (Fig. 7E). Overall, this suggests that WBI severely impairs the ability of memory CD4 T cells to clear the pathogen during secondary exposures.

## Discussion

The most common form of radiation exposure to humans is through RT to treat different forms of cancer.^3^ While RT is proven to be effective in treating various tumors, it also comes with the risk of adverse side effects.^6^^,^^7^ Because memory CD4 T cells play a critical role in providing direct and indirect help to other immune cells in clearing diverse pathogens,^17–20^^,^^26^^,^^28^^,^^29^ it is imperative to understand how radiation exposure directly affects these cells. Here, we use a SMARTA chimeric mouse model of LCMV infection to demonstrate that WBI causes long-term impairment in the maintenance and function of memory CD4 T cells.

Our group has already demonstrated that WBI impairs the maintenance and function of memory CD8 T cells. Naïve CD8 T cells experience a severe decline early after WBI and recover over time through HP and new thymic emigrants, whereas memory CD8 T cells sustain a long-lasting numerical decline.^14^^,^^16^^,^^38^ We demonstrate that this phenotype is also observed in CD4 T cells, where LCMV-Arm gp_61–80_ epitope–specific TCR-tg memory CD4 T cells sustain a severe numerical decline in PBLs, whereas naïve CD4 T cells recovered after 30 d of WBI. We also demonstrate that numerical decline early after WBI is seen in the blood, SLOs, and nonlymphoid organs. Previous studies using mouse models have shown that MPh CD4 T cells (CD44^+^) are more resistant than naïve CD4 T cells (CD44^−^) early after WBI.^10^^,^^55^ This is true in our hands as well, but when we examined the long-term kinetics of TCR-tg and endogenous polyclonal memory CD4 T cells in spleen, we observed that bona fide memory CD4 T cells sustained a long-term numerical decline. In PBLs, irradiated MPh CD4 T cells showed higher Ki-67 expression and recovery kinetics similar to those of naïve CD4 T cells, unlike TCR-tg memory CD4 T cells. This suggests that the endogenous MPh CD4 T cell compartment may be partially composed of virtual memory–like CD4 T cells. The existence of such virtual memory–like CD4 T cells has been demonstrated in uninfected SPF, germ-free, and antigen-free mice.^41–43^ However, in an infected host, the markers distinguishing virtual memory T cells from bona fide memory CD4 T cells remain to be identified. While HP is essential, thymic reconstitution has also been shown to play a critical role in the recovery of naïve CD4 and CD8 T cells following WBI.^38^^,^^48^ Unlike TCR-tg CD4 T cells, polyclonal virus-specific CD4 T cell precursors can be reconstituted in the thymus following WBI. Still, in the spleen, both TCR-tg and polyclonal memory CD4 T cell numbers were lower in WBI mice beyond 30 dpWBI. While TCR-tg cells help us understand the direct effects of WBI on pre-existing memory T cells that cannot be recovered, polyclonal memory CD4 T cells are translationally relevant for understanding the overall effect of WBI on memory CD4 T cells.

Unlike CD8 T cells, defining memory CD4 T cell subsets have not been straightforward, owing to their plasticity, heterogeneity, and functional diversity.^49^^,^^50^ Works from the past decade have established a PSGL1- and Ly6C-based approach to broadly define the Th1, T_CM_, and Tfh subsets in intracellular infection models through extensive phenotypic, transcriptional, and adoptive-transfer analyses.^35^^,^^36^^,^^39^^,^^49–51^ Also, there is evidence of effector and memory populations within these Th1 and Tfh subsets, as well as mature and precursor memory populations within the T_CM_ subset, depending on the time postinfection.^35^^,^^49^^,^^51^ Using this approach, we found that the TCR-tg and polyclonal memory CD4 T cells exhibit transient impairment in subset composition, which resolves over time. While deeper phenotypic and transcriptional analyses are required to definitively ascertain functional changes, we show that WBI causes fractional and per-cell-level changes in the expression of a few key markers within each subset. An increase in both the frequency and per-cell expression of PD-1 among Th1 and T_CM_ subsets following WBI potentially drives the persistent decline in the numbers of TCR-tg memory CD4 T cells. Although the fractions of CXCR3^+^ cells across all 3 subsets remain unaltered, WBI-driven per-cell expression decline in the Th1 and T_CM_ subsets could impair their migratory potential. WBI led to a decline in the frequency and per-cell expression of CD62L but only in the Th1 subset. However, CD62L is less stable and prone to shedding during in vitro assays; therefore, further characterization of CCR7 is critical, as it is a more reliable marker for identifying lymph node–homing CD4 T cells. Bcl-6 expression was unaltered across the subsets, with a trending increase in per-cell expression among Tfh cells following WBI. Although Bcl-6 is the lineage-defining transcription factor of Tfh cells, its expression is highest during the effector phase and declines at the late memory time point, paralleling the downregulation of PD-1.^56^^,^^57^ Therefore, the observed differences in per-cell Bcl-6 expression may not be biologically significant, and further characterization of Tfh-specific markers at this time point (such as CXCR5, FR4, CD127, and ICOS) is critical to understanding the effect of WBI on this subset. Furthermore, the inherent differences in survivorship potential among these subsets, dictated by effective DNA repair mechanisms, epigenetic regulation, and chromosomal access, and the effects of WBI on other immune cells that aid in their maintenance and survival, could also contribute to the subset and phenotypic changes observed and remain to be tested.

The key functions of primary memory T cells are to produce proinflammatory cytokines upon cognate antigen recognition, mount a robust secondary effector expansion, and ultimately clear the pathogen. We have previously shown that WBI impairs all of these functional aspects of primary memory CD8 T cells.^14^^,^^16^ We have shown that this impairment is even more pronounced among quaternary memory CD8 T cells generated through repetitive antigen recognition.^14^ However, studying the memory CD4 T cell response to repeated antigenic stimulation is difficult or impossible for several reasons. First, effector T cell expansion from naïve T cells in response to a pathogen is manyfold higher in CD8 T cells than in CD4 T cells, thereby generating fewer memory CD4 T cells than memory CD8 T cells.^58–61^ Additionally, studies of repetitive antigen stimulation to understand memory CD8 T cells have been enabled by technical and biological advances that are still lacking for CD4 T cells.^49^^,^^50^ Despite these shortcomings, we have successfully demonstrated that WBI regulates primary memory CD4 T cell function in vitro and in vivo. Both TCR-tg and endogenous memory CD4 T cells following WBI are impaired in their ability to produce proinflammatory cytokines in response to cognate antigen stimulation. This was true in both SPF mice infected with LCMV and the specific pathogen–experienced mouse model, in which animals are inherently in an inflammatory state prior to WBI exposure. We demonstrate that this translates to poor pathogen clearance in mice during a.LM-gp33+gp61 rechallenge. It is important to note that the host’s WBI status does not appear to influence the a.LM pathogen burden but does influence the a.LM-gp33+gp61 pathogen burden. Across these experiments, we demonstrated that the impairment is intrinsic to irradiated memory CD4 T cells, to the extent that they were unable to mount a robust secondary expansion in an unmanipulated recipient. Further studies assessing the genetic and epigenetic modifications in memory CD4 T cells following WBI are required to elucidate the drivers of these functional impairments. Also, T cell–extrinsic factors from WBI exposure, such as altered inflammation, immune cell status, and pathogenicity, remain to be tested.

In summary, our findings highlight the kinetics, subset composition, and functional impairment of memory CD4 T cells following WBI in a mouse model of LCMV-Arm infection. While the number of memory CD4 T cells declines early after WBI compared with 0 Gy mice, the gap continues to widen over time. These memory CD4 T cells sustain a transient imbalance in their subset composition early after WBI and exhibit phenotypic changes. WBI leads to per-cell-level cytokine dysfunction and secondary expansion of memory CD4 T cells during pathogen re-encounter, thereby impeding the host’s ability to clear the pathogen. These findings inform future work aimed at improving the memory CD4 T cell response to diverse pathogens and vaccines in patients with a history of radiation exposure.

## Supplementary Material

vkag244_Supplementary_Data

## Data Availability

All data associated with this study can be found in the main text or the Supplementary Materials.
