# Supplementary material for: Virus-specific memory CD4 T cells sustain long-term numerical and functional impairment following whole-body irradiation
Source: J Immunol. 2026 Sep 10;215(9):vkag244. doi: 10.1093/jimmun/vkag244 (PMC13561510; doi:10.1093/jimmun/vkag244)
Supplement: Supplementary file 1 [file vkag244_supplementary_data.docx]

**Figure S1**. **Memory CD4 T cells are susceptible to WBI across lymphoid and non-lymphoid tissues.** Representative flow plots (Left) and the number (Right) of Naïve, TCR-tg memory, and MPh CD4 T cells in the mesenteric lymph nodes (A), femur (B), and kidney (C) of mice 8 days post-WBI (dpWBI). Data are representative of 2 independent experiments with 3 to 4 mice per group. In A-C, the fold change between the groups is highlighted. *P* values in A-C were derived from two-tailed unpaired Student’s t-tests. All graphs show the mean ± SEM.

**Figure S2. Memory CD4 T cell subsets in the circulation and lymphoid tissues skew toward Th1 and away from Tfh early after WBI.** (A) Experimental design – naïve TCR-tg SMARTA CD4 T cells were transferred into congenically distinct C57BL/6 mice, followed by LCMV-Arm infection the next day. Three weeks after infection, mice were subjected to 5Gy or mock (0Gy) WBI, and the tissues were harvested 8 dpWBI. (B) Representative gating- After gating into TCR-tg memory and endogenous MPh CD4 T cells, Th1 (Ly6C^hi^PSGL-1^+^), T_CM_ (Ly6C^lo^PSGL-1^+^), and TfH (Ly6C^lo^PSGL-1^-^) subsets were identified. Frequency of TCR-tg (top) and MPh (bottom) CD4 T cells that are Th1, T_CM_, and Tfh in PBLs (C), Spleen (D), and mesenteric LNs (E) are compared between 0Gy and 5Gy mice. Data are representative of 2 independent experiments with 3 to 4 mice per group. *P* values in C, D, and E were derived from two-way ANOVA with Sidak’s multiple comparisons test. All graphs show the mean ± SEM. Graphical illustration (A) was created with [BioRender.com](http://biorender.com/).

**Figure S3. WBI transiently alters the subset composition of the TCR-tg and polyclonal memory CD4 T cell subsets.** (A) Experimental design – Upon generating TCR-tg memory CD4 T cell chimeric mice, they were subjected to 5Gy or mock (0Gy) WBI, and the spleen was harvested 33 dpWBI. Representative flow plots (B) and the frequency (C) of Th1, T_CM_, and Tfh cells among TCR-tg memory CD4 T cell subsets in the spleen of mice described in A. (D) Experimental design – Upon generating virus-specific polyclonal memory CD4 T cells in mice lacking TCR-tg cells, they were subjected to 5Gy or mock (0Gy) WBI, and the spleen was harvested 12 and 33 dpWBI. Representative flow plots (E, G) and the frequency (F, H) of Th1, T_CM_, and Tfh cells among GP_66-77_-tetramer-specific polyclonal memory CD4 T cells in the spleen at 12 and 33 dpWBI, in mice described in D. Data are representative of 2 independent experiments with 4 to 5 mice per group. *P* values were derived from two-way ANOVA with Sidak’s multiple comparisons test. All graphs show the mean ± SEM. Graphical illustration (A and D) were created with [BioRender.com](http://biorender.com/).

**Figure S4. WBI alters the frequency of memory CD4 T cell subsets expressing CXCR3, CD62L, Bcl-6, and PD-1, as well as their per-cell expression.** The data are analyzed from TCR-tg memory CD4 T cells at 33 dpWBI, isolated from the spleen of mice described in Fig. S3A. Representative histogram and the gMFI of CXCR3 (A), CD62L (B), Bcl-6 (C), and PD-1 (D) among Th1, T_CM_, and Tfh subsets of TCR-tg memory CD4 T cells. Data are representative of 2 independent experiments with 4 to 5 mice per group. *P* values were derived from two-way ANOVA with Sidak’s multiple comparisons test. All graphs show the mean ± SEM.

**Figure S5. WBI-mediated cytokine dysfunction is intrinsic to memory CD4 T cells.** (A) Experimental design – Upon generating TCR-tg memory CD4 T cell chimeric mice, they were subjected to 5Gy or mock (0Gy) WBI, and the spleen was harvested 30 dpWBI. (B) Graphical description of CFSE labeling of 0Gy splenocytes and co-culture with an equal number of 5Gy splenocytes before stimulation with gp_61-80_ peptide. (C) Representative flow plots and the gating strategy used to find IFNγ, TNFα, and IL-2 expression among TCR-tg memory CD4 T cells from 0Gy (CFSE+) and 5Gy (CFSE-) hosts upon 0.5 µg/mL gp_61-80_ peptide stimulation. The frequency of TCR-tg memory CD4 T cells producing single cytokines (D), and triple cytokines (E), namely IFNγ, TNFα, and IL-2. Data are representative of 2 independent experiments with 4 mice per group. *P* values in D and E were derived from two-way ANOVA with Sidak’s multiple comparisons test. All graphs show the mean ± SEM. Graphical illustration (A) was created with [BioRender.com](http://biorender.com/).
